# Supplementary material for: Unveiling the key factor for the phase reconstruction and exsolved metallic particle distribution in perovskites
Source: Nat Commun. 2021 Nov 24;12:6814. doi: 10.1038/s41467-021-26739-1 (PMC8613209; doi:10.1038/s41467-021-26739-1)
Supplement: Supplementary file 1 — Supplementary Information [file 41467_2021_26739_MOESM1_ESM.pdf]

Supplementary Information

Unveiling the key factor for the phase reconstruction and exsolved metallic particle distribution in perovskites

Hyunmin Kim[1,6], Chaesung Lim[2,6], Ohhun Kwon[3,6], Jinkyung Oh[1], Matthew T. Curnan[2], Hu Young Jeong[4], Sihyuk Choi[5,], Jeong Woo Han[2,*] and Guntae Kim[1,*]*

[1] School of Energy and Chemical Engineering, Ulsan National Institute of Science and Technology (UNIST), Ulsan, 44919, Republic of Korea

[2] Department of Chemical Engineering, Pohang University of Science and Technology (POSTECH), Pohang 37673, Republic of Korea

[3] Department of Chemical and Biomolecular Engineering, University of Pennsylvania, Philadelphia, Pennsylvania 19104, United States of America.

[4] Department of Materials Science and Engineering and UNIST Central Research Facilities (UCRF), Ulsan National Institute of Science and Technology (UNIST), Ulsan, 44919, Republic of Korea

[5] Department of Mechanical Engineering (Aeronautics, Mechanical and Electronic Convergence Engineering), Kumoh National Institute of Technology, Gyeongbuk 39177, Republic of Korea.

[6] These authors contributed equally to this work.

---

* Corresponding authors.

Email: sh.choi@kumoh.ac.kr (S.C.), jwhan@postech.ac.kr (J.W.H.), gtkim@unist.ac.kr (G.K.).

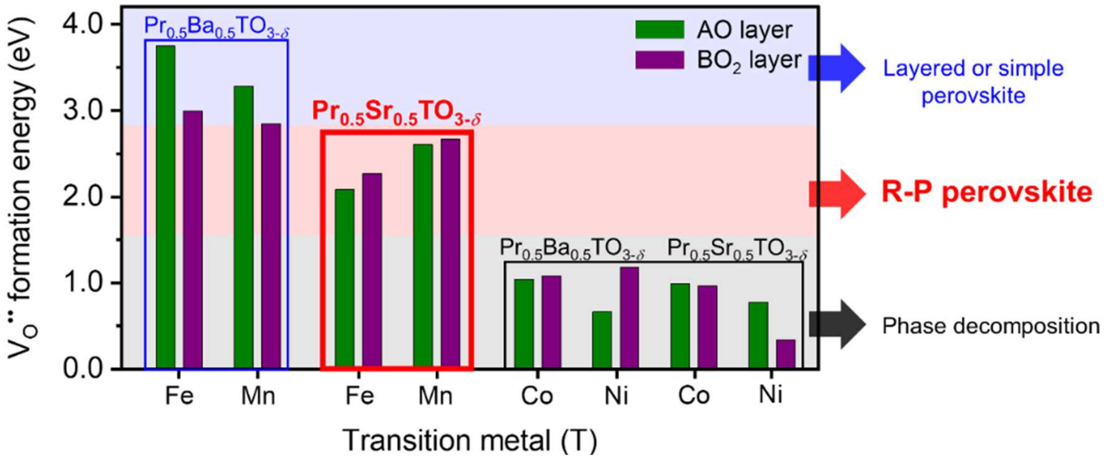

Supplementary Figure 1. Oxygen vacancy formation calculations. Calculated oxygen vacancy formation energies of $Pr_{0.5}(Ba/Sr)_{0.5}TO_{3-\delta}$ (T = Mn, Fe, Co, and Ni) from the surface AO (green bar) and BO_2 (purple bar) networks and the predicted phase change under reducing condition. Note that the Gibbs free energy for the oxygen vacancy formation ($G_{vf\text{-}O}$) was additionally calculated to contain the temperature and oxygen partial pressure factors in the $E_{vf\text{-}O}$ calculations.

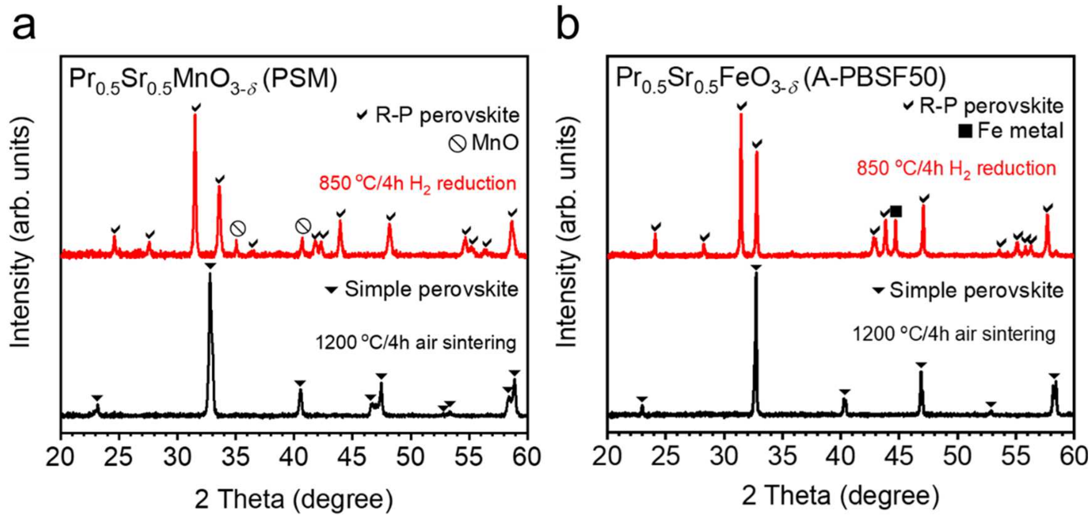

Supplementary Figure 2. X-ray diffraction (XRD) patterns of $Pr_{0.5}Sr_{0.5}TO_{3-\delta}$ (T = Mn and Fe) before and after reduction. (a – b) XRD patterns of (a) $Pr_{0.5}Sr_{0.5}MnO_{3-\delta}$ sintered at 1200 ^oC for 4 hours in air atmosphere (black) and reduced at 850 ^oC for 4 hours in humidified H_2 atmosphere (3% H_2O, red) and (b) $Pr_{0.5}Sr_{0.5}FeO_{3-\delta}$ (A-PBSF50) sintered at 1200 ^oC for 4 hours in air atmosphere (black) and reduced at 850 ^oC for 4 hours in humidified H_2 atmosphere (3% H_2O, red).

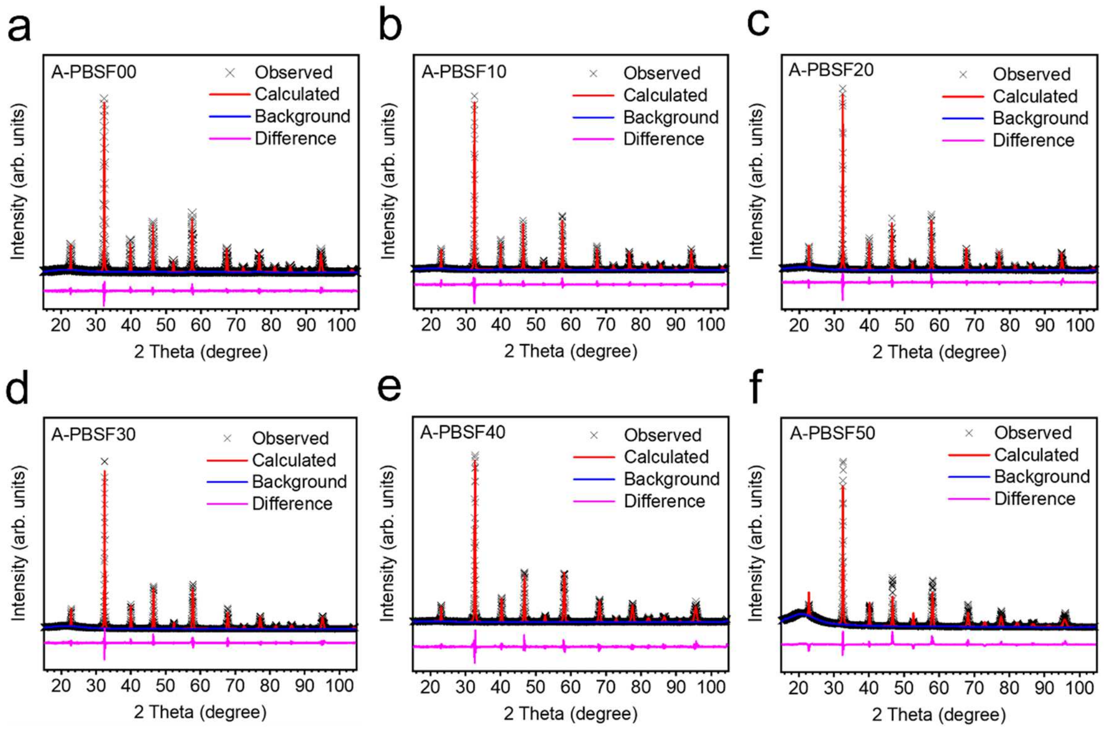

Supplementary Figure 3. X-ray diffraction (XRD) Rietveld refinement profiles of air-sintered $Pr_{0.5}Ba_{0.5-x}Sr_xFeO_{3-\delta}$ material. XRD Rietveld refinement profiles of $Pr_{0.5}Ba_{0.5-x}Sr_xFeO_{3-\delta}$ material sintered at 1200 °C for 4 hours in air atmosphere: (a) A-PBSF00 (x = 0), (b) A-PBSF10 (x = 0.1), (c) A-PBSF20 (x = 0.2), (d) A-PBSF30 (x = 0.3), (e) A-PBSF40 (x = 0.4), and (f) A-PBSF50 (x = 0.5). All samples possess same simple perovskite structure with space group P*m*-3*m*.

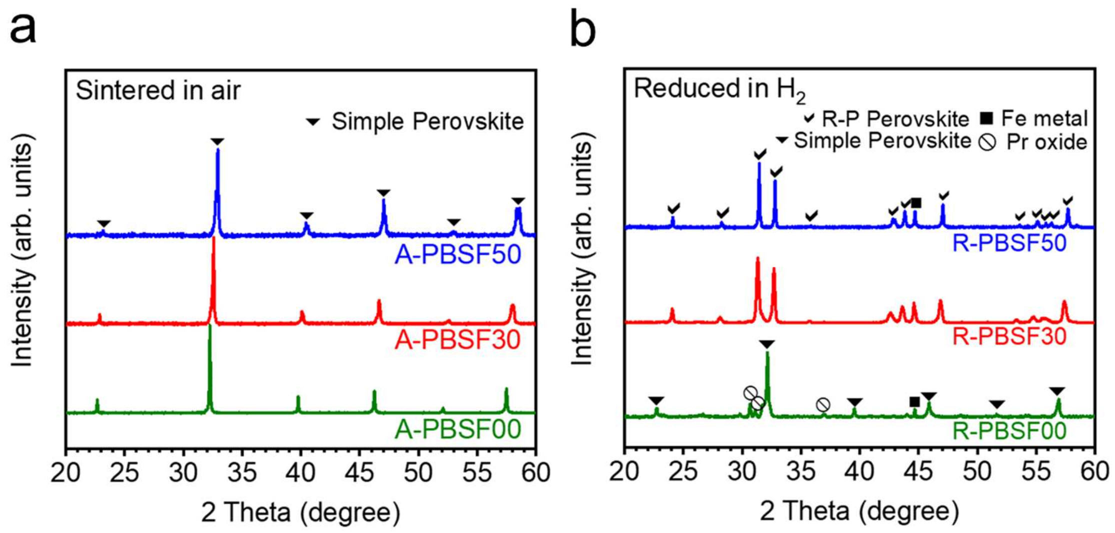

Supplementary Figure 4. X-ray diffraction (XRD) patterns of $Pr_{0.5}Ba_{0.5-x}Sr_xFeO_{3-\delta}$ (x = 0, 0.3 and 0.5) before and after reduction. (a – b) XRD patterns of $Pr_{0.5}Ba_{0.5-x}Sr_xFeO_{3-\delta}$ (x = 0, 0.3 and 0.5) (a) sintered at 1200 °C for 4 hours in air atmosphere and (b) reduced at 850 °C for 4 hours in H_2 atmosphere.

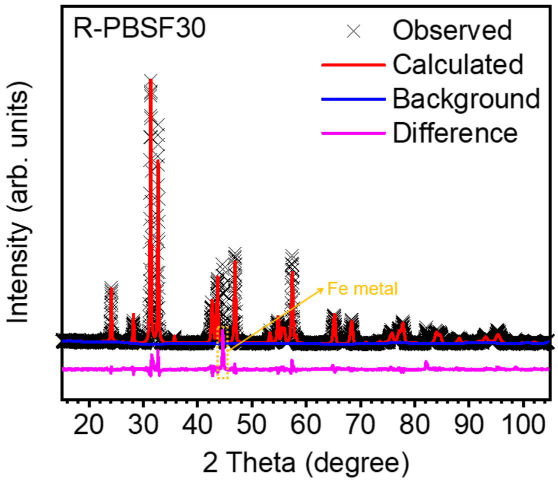

Supplementary Figure 5. X-ray diffraction (XRD) Rietveld refinement data of $(Pr_{0.5}Ba_{0.2}Sr_{0.3})_2FeO_{4+\delta}$ – Fe metal (R-PBSF30). XRD Rietveld refinement data of R-PBSF30 reduced at 850 °C for 4 hours in humidified H_2 atmosphere (3% H_2O). The R-PBSF30 exhibited a tetragonal structure, space group I4/*mmm* with lattice parameters of a = b = 3.879 and c = 12.704 Å, which is clearly different to the simple perovskite $Pr_{0.5}Ba_{0.2}Sr_{0.3}FeO_{3-\delta}$ (A-PBSF30) with space group P*m*-3*m* (**Supplementary Fig. 3d**).

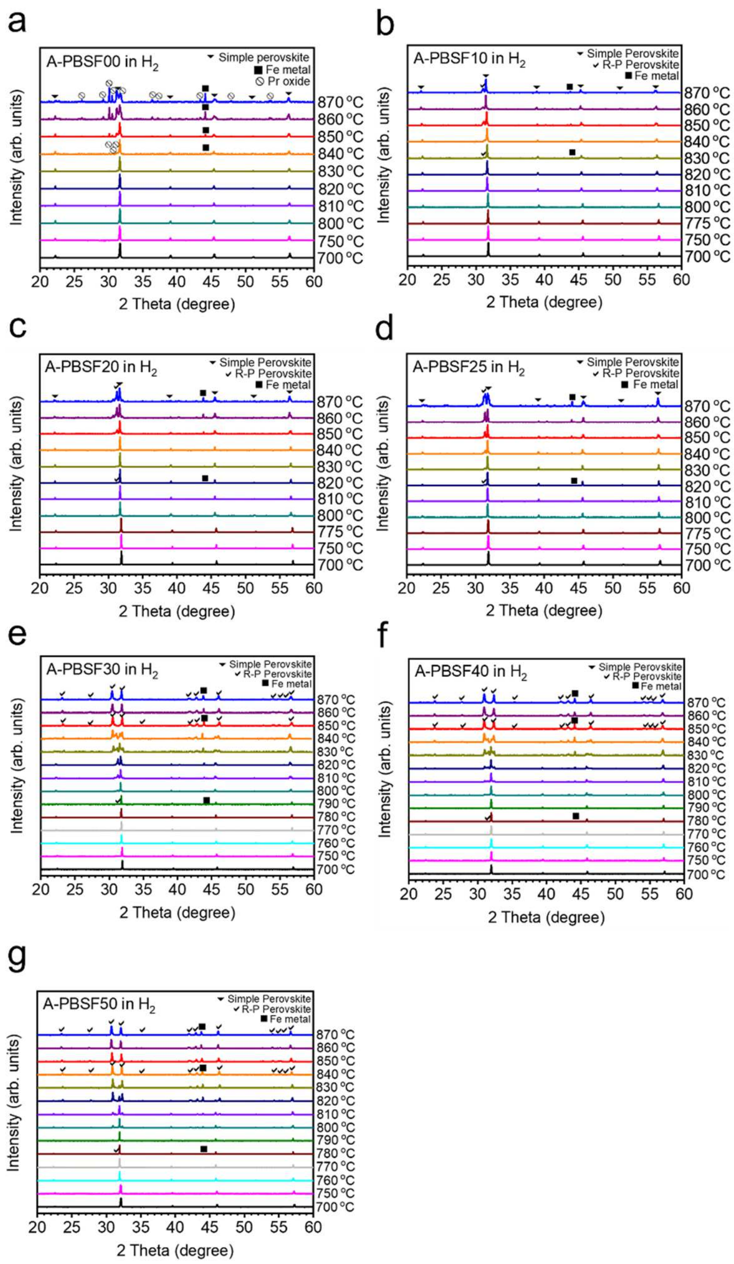

Supplementary Figure 6. ***In-situ*** **powder X-ray diffraction (XRD) patterns of $Pr_{0.5}Ba_{0.5-x}Sr_xFeO_{3-\delta}$ material.** (a) A-PBSF00 (x = 0), (b) A-PBSF10 (x = 0.1), (c) A-PBSF20 (x = 0.2), (d) A-PBSF25 (x = 0.25), (e) A-PBSF30 (x = 0.3), (f) A-PBSF40 (x = 0.4), and (g) A-PBSF50 (x = 0.5). The XRD measurements were conducted from 700 °C to 870 °C under H_2 environment (3% H_2O).

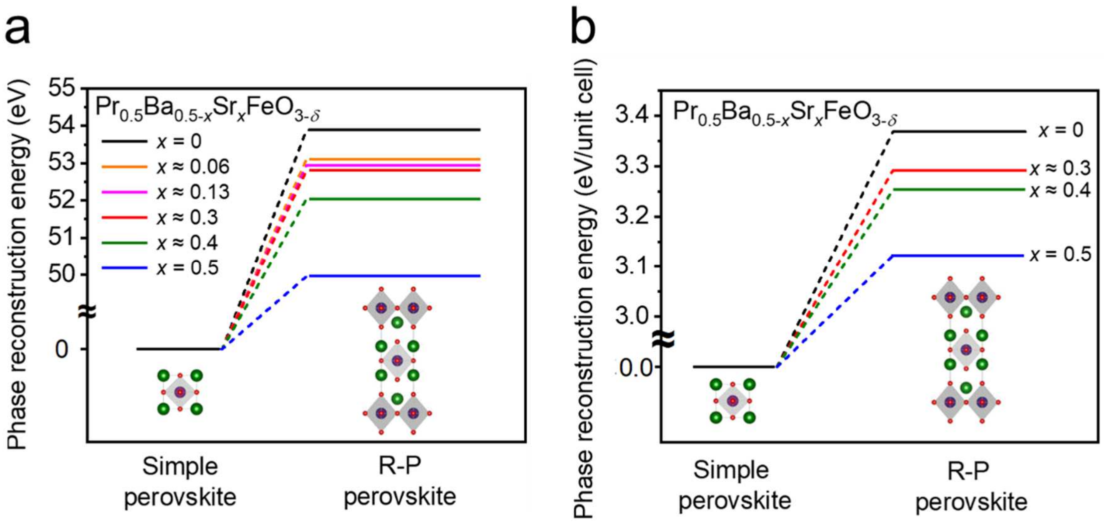

Supplementary Figure 7. Phase reconstruction energy calculations. (a – b) Calculated profiles of the relative total energy required for the phase reconstruction from simple perovskite to R-P perovskite as a function of Sr^{2+} concentration (a) in six models in terms of eV and (b) in four models in terms of eV/unit cell.

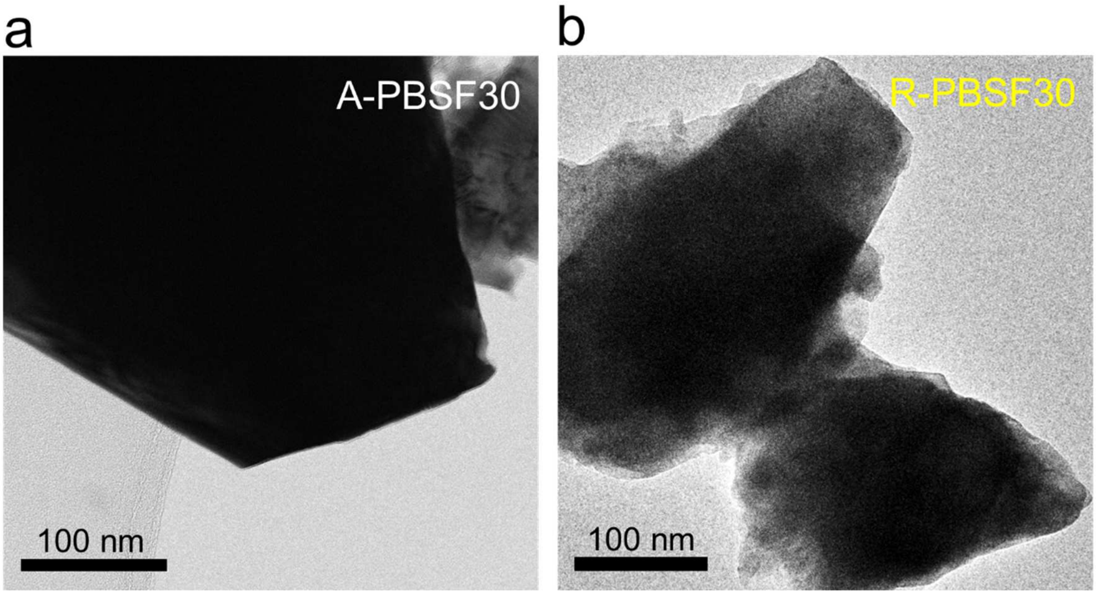

Supplementary Figure 8. Bright-field transmission electron microscope (BF-TEM) images of $Pr_{0.5}Ba_{0.2}Sr_{0.3}FeO_{3-\delta}$ before and after reduction. (a – b) BF-TEM images of (a) $Pr_{0.5}Ba_{0.2}Sr_{0.3}FeO_{3-\delta}$ (A-PBSF30) sintered at 1200 ^{o}C for 4 hours in air atmosphere and (b) $(Pr_{0.5}Ba_{0.2}Sr_{0.3})_2FeO_{4+\delta}$ – Fe metal (R-PBSF30) reduced at 850 ^{o}C for 4 hours in humidified H_2 environment (3% H_2O).

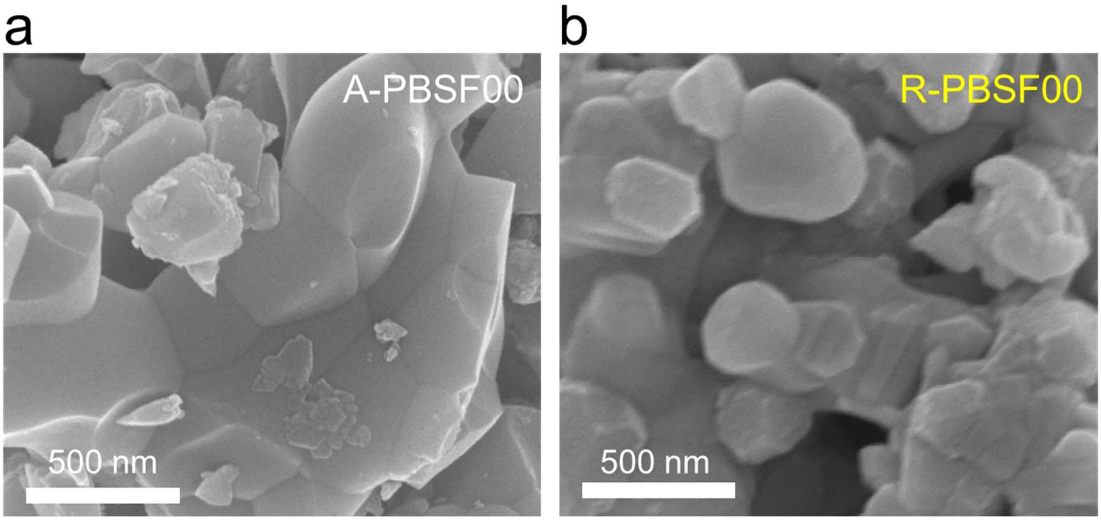

Supplementary Figure 9. Scanning electron microscope (SEM) images of $Pr_{0.5}Ba_{0.5}FeO_{3-\delta}$ before and after reduction. SEM images presenting the morphologies of (a) $Pr_{0.5}Ba_{0.5}FeO_{3-\delta}$ (A-PBSF00) sintered at 1200 °C for 4 hours in air atmosphere and (b) $Pr_{0.5}Ba_{0.5}FeO_{3-\delta}$ – Fe metal & Pr oxide (R-PBSF00) reduced at 850 °C for 4 hours in humidified H_2 environment (3% H_2O).

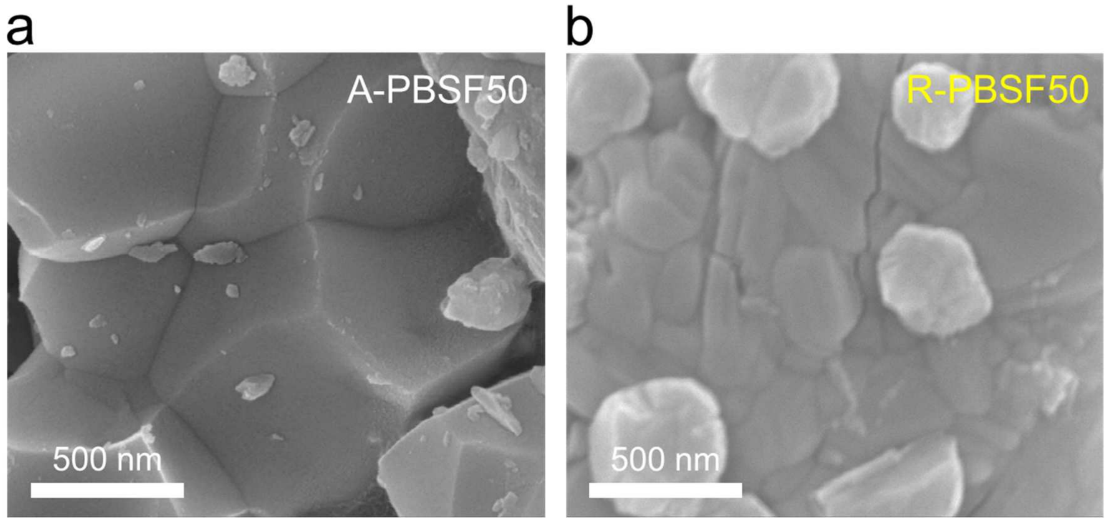

Supplementary Figure 10. Scanning electron microscope (SEM) images of $\mathbf{Pr_{0.5}Sr_{0.5}FeO_{3-\delta}}$ before and after reduction. SEM images presenting the morphologies of (a) $Pr_{0.5}Sr_{0.5}FeO_{3-\delta}$ (A-PBSF50) sintered at 1200 °C for 4 hours in air atmosphere and (b) $Pr_{0.5}Sr_{0.5}FeO_{3-\delta}$ – Fe metal (R-PBSF50) reduced at 850 °C for 4 hours in humidified H_2 environment (3% H_2O).

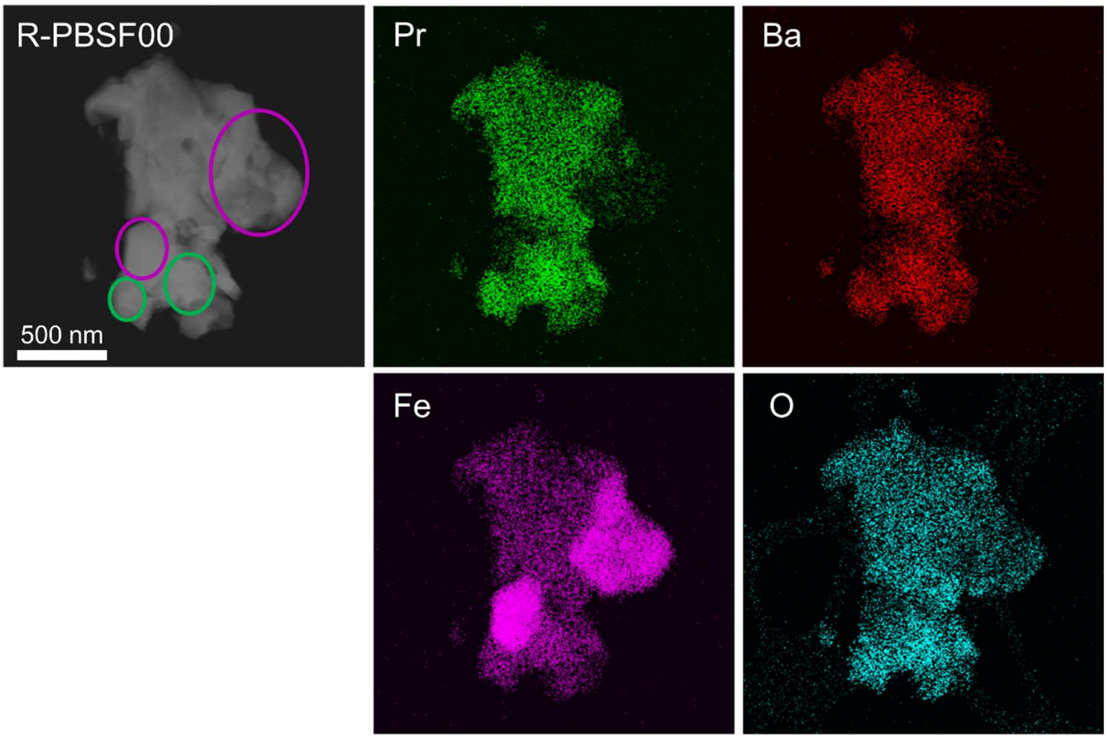

Supplementary Figure 11. High-angle annular dark field (HAADF) image of reduced $Pr_{0.5}Ba_{0.5}FeO_{3-\delta}$ – Fe metal & Pr oxide (R-PBSF00). HAADF image of R-PBSF00 sintered at 1200 °C for 4 hours in air environment and reduced at 850 °C for 4 hours in humidified H_2 environment and the elemental mapping of Pr, Ba, Fe and O, respectively. Both Pr oxide and Fe metal particles (about 400 to 500 nm for Pr oxide and 600 to 800 nm for Fe metal) were segregated onto the surface of R-PBSF00 after reduction in H_2.

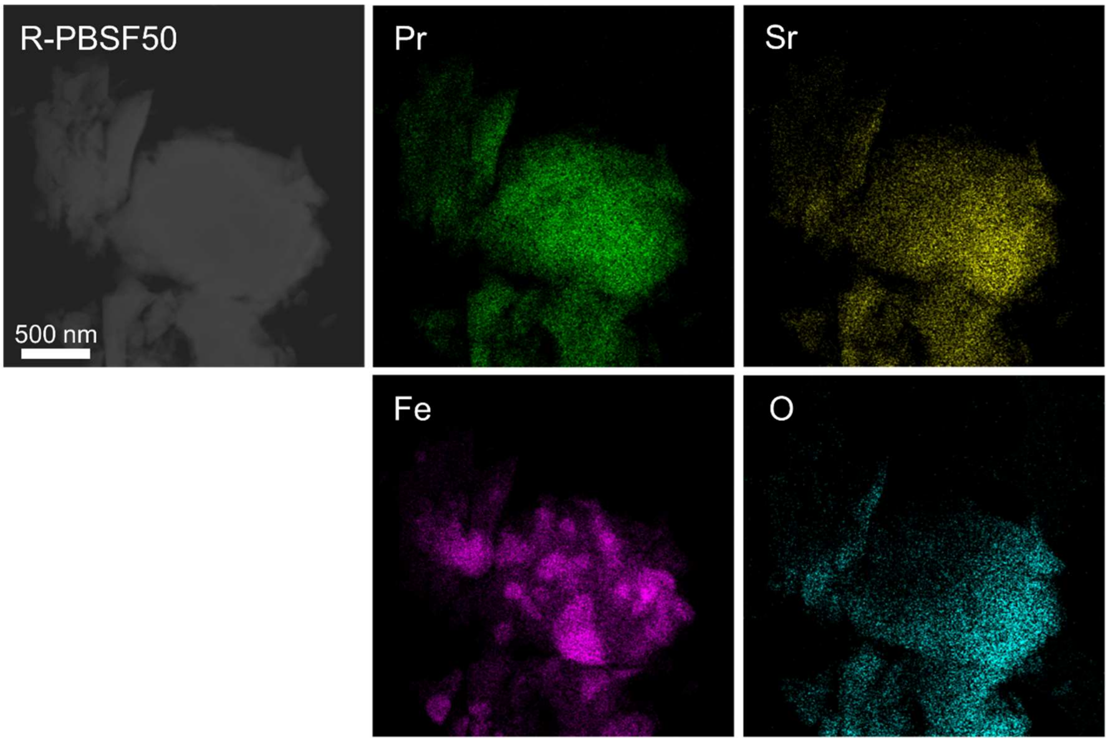

Supplementary Figure 12. High-angle annular dark field (HAADF) image of reduced $Pr_{0.5}Ba_{0.5}FeO_{3-\delta}$ – Fe metal (R-PBSF50). HAADF image of R-PBSF50 sintered at 1200 °C for 4 hours in air environment and reduced at 850 °C for 4 hours in humidified H_2 environment and the elemental mapping of Pr, Sr, Fe and O, respectively. Only Fe metal particles (about 400 to 600 nm) were segregated onto the surface of R-PBSF50 after reduction in H_2.

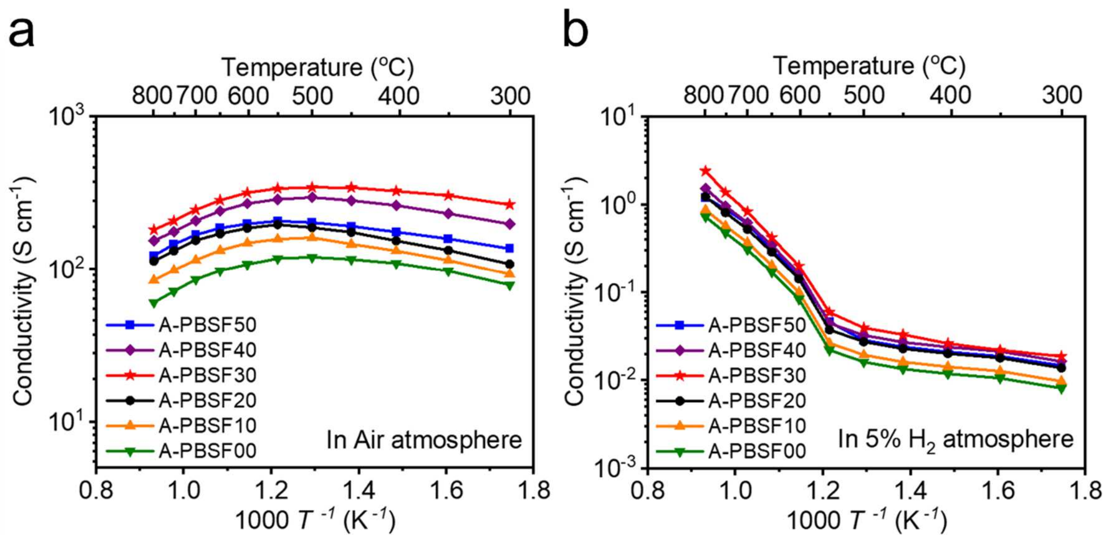

Supplementary Figure 13. Electrical conductivity measurements. Electrical conductivity measurements of $Pr_{0.5}Ba_{0.5-x}Sr_xFeO_{3-\delta}$ (x = 0, 0.1, 0.2, 0.3, 0.4 and 0.5) with respect to temperature in (a) air and (b) 5% H_2 atmospheres.

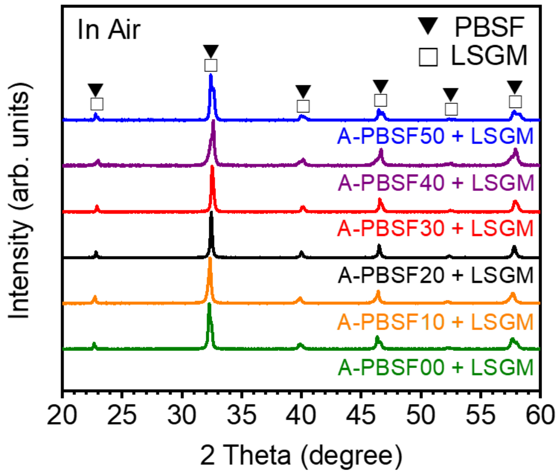

Supplementary Figure 14. Thermo-chemical compatibility test. X-ray diffraction patterns of $Pr_{0.5}Ba_{0.5-x}Sr_xFeO_{3-\delta}$ (x = 0, 0.1, 0.2, 0.3, 0.4 and 0.5)- $La_{0.9}Sr_{0.1}Ga_{0.8}Mg_{0.2}O_{3-\delta}$ (LSGM) composites sintered at 950 °C for 4 hours in air atmosphere, *i.e.*, cell fabrication temperature. $Pr_{0.5}Ba_{0.5-x}Sr_xFeO_{3-\delta}$ (x = 0, 0.1, 0.2, 0.3, 0.4 and 0.5)-LSGM composites were mixed with weight ratio 1:1.

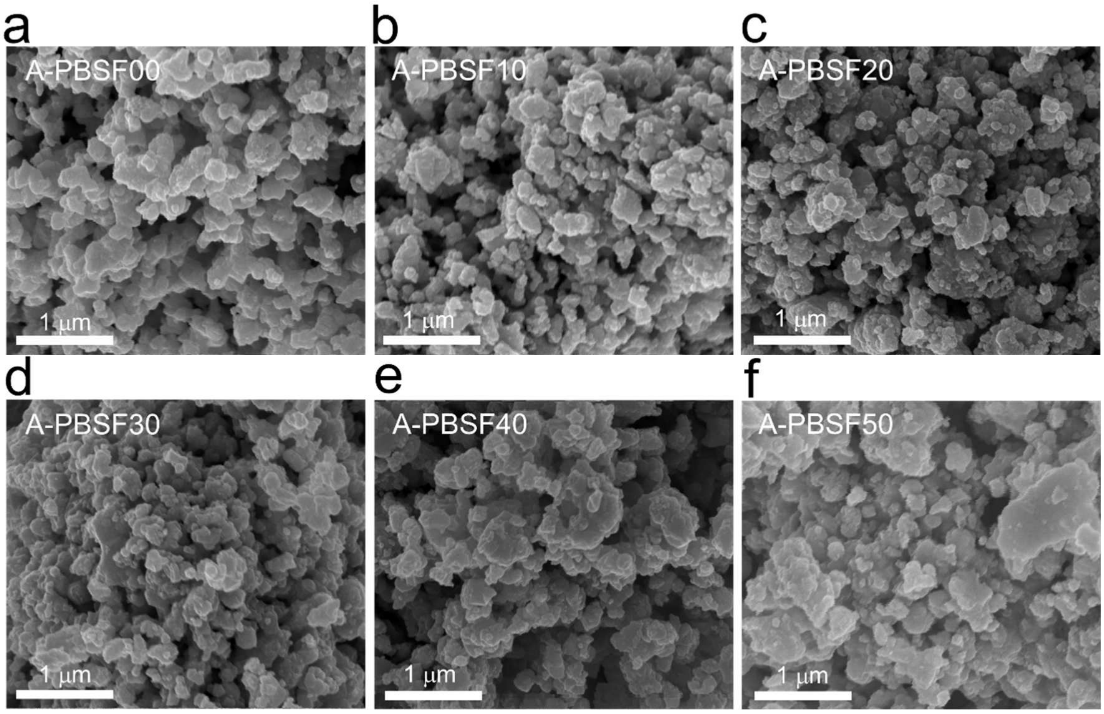

Supplementary Figure 15. Surface morphology evaluation of air-sintered $Pr_{0.5}Ba_{0.5-x}Sr_xFeO_{3-\delta}$ materials. Scanning electron microscope (SEM) images showing typical microstructures of $Pr_{0.5}Ba_{0.5-x}Sr_xFeO_{3-\delta}$ materials sintered at 950 °C for 4 hours in air atmosphere: (a) A-PBSF00 (x = 0), (b) A-PBSF10 (x = 0.1), (c) A-PBSF20 (x = 0.2), (d) A-PBSF30 (x = 0.3), (e) A-PBSF40 (x = 0.4), and (f) A-PBSF50 (x = 0.5).

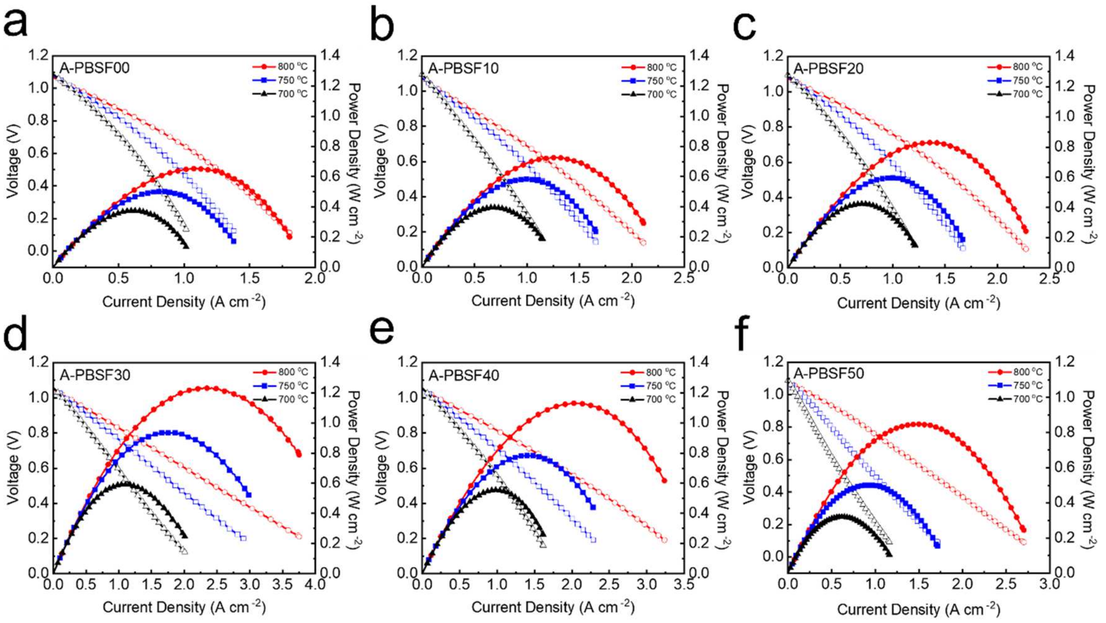

Supplementary Figure 16. Electrochemical performance measurements of $Pr_{0.5}Ba_{0.5-x}Sr_xFeO_{3-\delta}$ symmetrical cells. I-V curves and corresponding power densities of symmetrical cells with $Pr_{0.5}Ba_{0.5-x}Sr_xFeO_{3-\delta}$ electrodes using humidified H_2 (3% H_2O) as fuel and air as oxidant from 700 °C to 800 °C with intervals of 50 °C: (a) A-PBSF00 (x = 0), (b) A-PBSF10 (x = 0.1), (c) A-PBSF20 (x = 0.2), (d) A-PBSF30 (x = 0.3), (e) A-PBSF40 (x = 0.4), and (f) A-PBSF50 (x = 0.5).

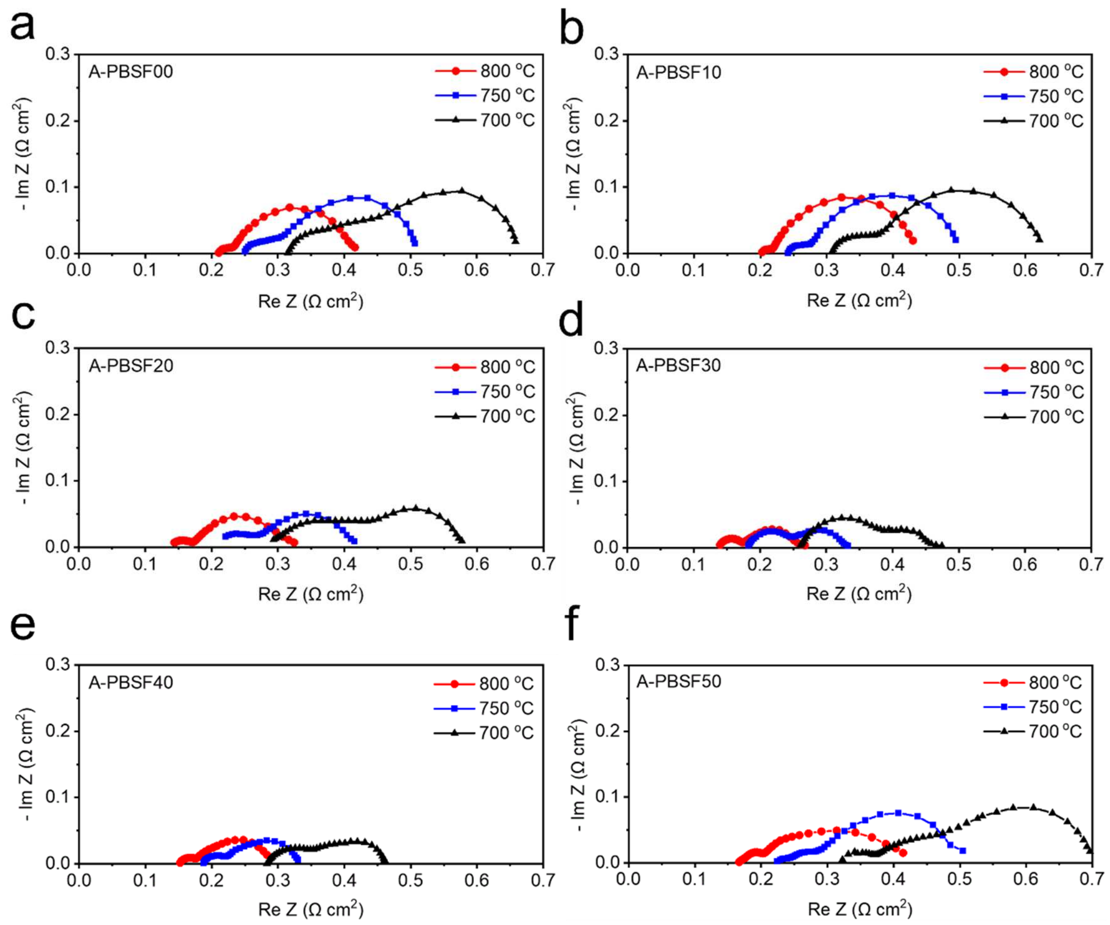

Supplementary Figure 17. Electrochemical impedance spectra (EIS) of $Pr_{0.5}Ba_{0.5-x}Sr_xFeO_{3-\delta}$ symmetrical cells. (a – f) EIS of the symmetrical cells with $Pr_{0.5}Ba_{0.5-x}Sr_xFeO_{3-\delta}$ electrodes using humidified H_2 (3% H_2O) as fuel and air as oxidant from 700 °C to 800 °C with intervals of 50 °C: (a) A-PBSF00 (x = 0), (b) A-PBSF10 (x = 0.1), (c) A-PBSF20 (x = 0.2), (d) A-PBSF30 (x = 0.3), (e) A-PBSF40 (x = 0.4), and (f) A-PBSF50 (x = 0.5).

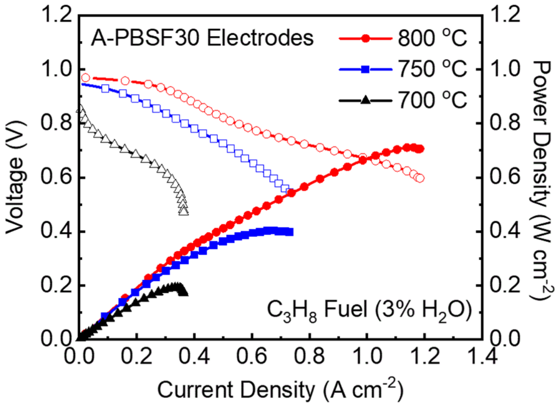

Supplementary Figure 18. Electrochemical performance evaluation of $Pr_{0.5}Ba_{0.2}Sr_{0.3}FeO_{3-\delta}$ symmetrical cell under C_3H_8 fuel. I-V curves and the corresponding power densities of symmetrical cells with $Pr_{0.5}Ba_{0.2}Sr_{0.3}FeO_{3-\delta}$ (A-PBSF30) electrodes using humidified C_3H_8 (3% H_2O) as fuel and air as oxidant from 700 °C to 800 °C with intervals of 50 °C.

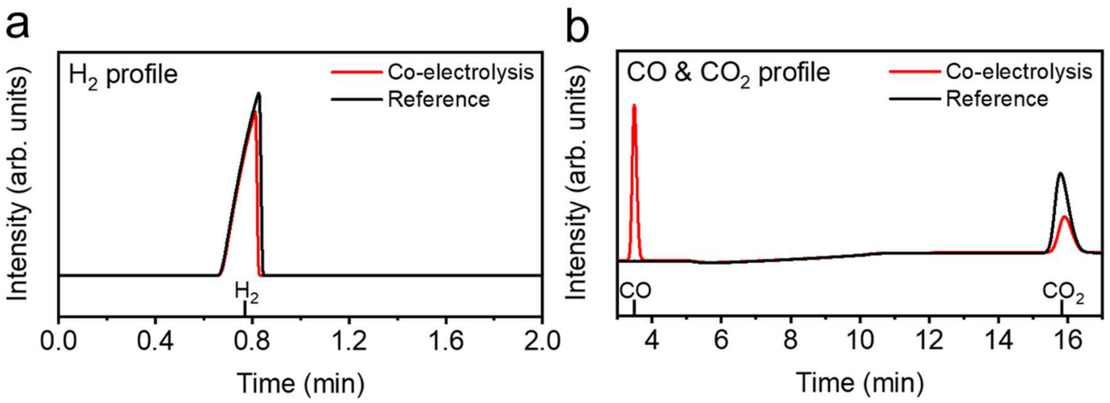

Supplementary Figure 19. Gas chromatography (GC) measurements. (a – b) The *in-operando* qualitative gas GC profiles of (a) H_2 gas and (b) CO & CO_2 gases before (Reference, black) and during co-electrolysis at 800 ^{o}C and 1.5 V for the $Pr_{0.5}Ba_{0.2}Sr_{0.3}FeO_{3-\delta}$ (A-PBSF30) symmetrical cell (Co-electrolysis, red). The quantitative analysis of the generated gas products (H_2 & CO) were determined by the additional measurement of the actual flow rate of the reference gas and the outlet gas during the co-electrolysis. The exact volume of gases were calibrated through a bubble flow-meter.

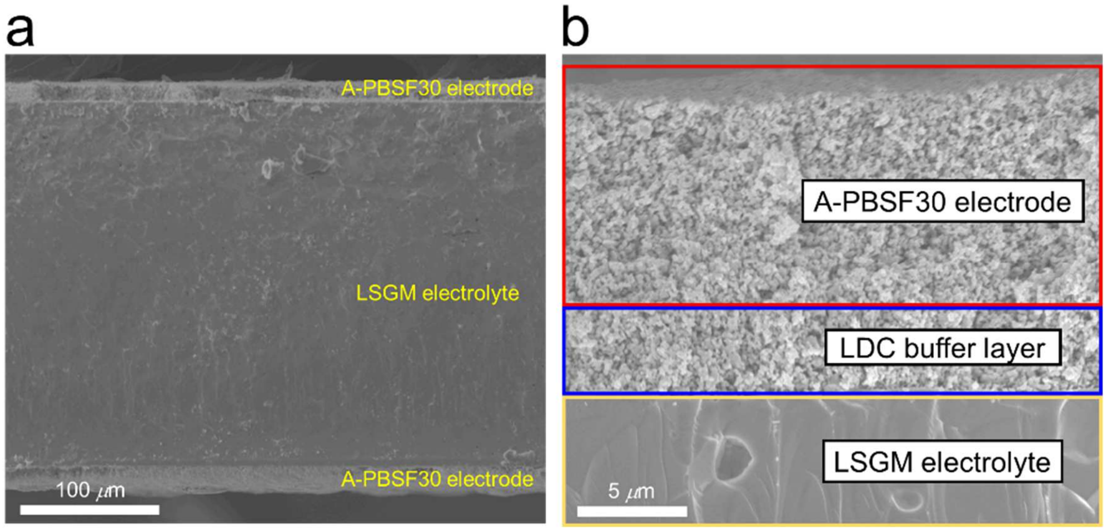

Supplementary Figure 20. Cross-sectional scanning electron microscopy (SEM) images. (a) Cross-sectional SEM image of $Pr_{0.5}Ba_{0.2}Sr_{0.3}FeO_{3-\delta}$ (A-PBSF30) symmetrical cell with cell configuration of A-PBSF30|$La_{0.4}Ce_{0.6}O_{2-\delta}$ (LDC)|$La_{0.9}Sr_{0.1}Ga_{0.8}Mg_{0.2}O_{3-\delta}$ (LSGM)|LDC|A-PBSF30 sintered at 950 °C for 4 hours in air. (b) SEM image displaying the microstructure of the interface between electrode/buffer layer and the electrolyte.

Supplementary Table 1. Chemical composition and abbreviation of specimens.

Composition	Abbreviation
$Pr_{0.5}Ba_{0.5}FeO_{3-\delta}$	A-PBSF00
$Pr_{0.5}Ba_{0.4}Sr_{0.1}FeO_{3-\delta}$	A-PBSF10
$Pr_{0.5}Ba_{0.3}Sr_{0.2}FeO_{3-\delta}$	A-PBSF20
$Pr_{0.5}Ba_{0.25}Sr_{0.25}FeO_{3-\delta}$	A-PBSF25
$Pr_{0.5}Ba_{0.2}Sr_{0.3}FeO_{3-\delta}$	A-PBSF30
$Pr_{0.5}Ba_{0.1}Sr_{0.4}FeO_{3-\delta}$	A-PBSF40
$Pr_{0.5}Sr_{0.5}FeO_{3-\delta}$	A-PBSF50
$Pr_{0.5}Ba_{0.5}FeO_{3-\delta}$ – Fe metal & Pr oxide<br>(after reduction in H_2)	R-PBSF00
$(Pr_{0.5}Ba_{0.2}Sr_{0.3})_2FeO_{4+\delta}$ – Fe metal<br>(after reduction in H_2)	R-PBSF30
$(Pr_{0.5}Sr_{0.5})_2FeO_{4+\delta}$ – Fe metal<br>(after reduction in H_2)	R-PBSF50
$Pr_{0.5}Ba_{0.5-x}Sr_xFeO_{3-\delta}$ (x = 0, 0.1, 0.2, 0.3, 0.4 and 0.5)	PBSF

Supplementary Table 2. X-ray diffraction (XRD) Rietveld refinement data of $Pr_{0.5}Ba_{0.5-x}Sr_xFeO_{3-\delta}$ (x = 0, 0.1, 0.2, 0.3, 0.4 and 0.5) displaying space group, lattice parameters and unit cell volumes.

Composition	Space Group	Cell Parameter (Å)	Cell Volume (Å^3)
A-PBSF00 (*x* = 0)	P*m*-3*m* (Cubic)	3.944 (a = b = c)	61.35
A-PBSF10 (*x* = 0.1)	P*m*-3*m* (Cubic)	3.934 (a = b = c)	60.88
A-PBSF20 (*x* = 0.2)	P*m*-3*m* (Cubic)	3.920 (a = b = c)	60.24
A-PBSF30 (*x* = 0.3)	P*m*-3*m* (Cubic)	3.906 (a = b = c)	59.59
A-PBSF40 (*x* = 0.4)	P*m*-3*m* (Cubic)	3.897 (a = b = c)	59.18
A-PBSF50 (*x* = 0.5)	P*m*-3*m* (Cubic)	3.892 (a = b = c)	58.68

Supplementary Table 3. Calculated Goldschmidt's factors for $Pr_{0.5}Ba_{0.5-x}Sr_xFeO_{3-\delta}$ (x = 0, 0.1, 0.2, 0.3, 0.4 and 0.5).

Composition	Goldschmidt's tolerance factor
A-PBSF00 (x = 0)	0.95
A-PBSF10 (x = 0.1)	0.94
A-PBSF20 (x = 0.2)	0.94
A-PBSF30 (x = 0.3)	0.93
A-PBSF40 (x = 0.4)	0.92
A-PBSF50 (x = 0.5)	0.91

The below equation was used to calculate the Goldschmidt tolerance factor for $Pr_{0.5}Ba_{0.5-x}Sr_xFeO_{3-\delta}$ materials (x = 0, 0.1, 0.2, 0.3, 0.4 and 0.5), where R_A, R_B and R_O are ionic radius of A-cation, radius of B-cation and radius of oxygen anion, respectively. The Goldschmidt tolerance factors for $Pr_{0.5}Ba_{0.5-x}Sr_xFeO_{3-\delta}$ materials were 0.95, 0.94, 0.94, 0.93, 0.92, and 0.91 for A-PBSF00 (x = 0), A-PBSF10 (x = 0.1), A-PBSF20 (x = 0.2), A-PBSF30 (x = 0.3), A-PBSF40 (x = 0.4), and A-PBSF50 (x = 0.5), respectively. All $Pr_{0.5}Ba_{0.5-x}Sr_xFeO_{3-\delta}$ (x = 0, 0.1, 0.2, 0.3, 0.4 and 0.5) revealed Goldschmidt tolerance factor between 0.9 to 1 ($0.9 < t < 1$), representing all $Pr_{0.5}Ba_{0.5-x}Sr_xFeO_{3-\delta}$ (x = 0, 0.1, 0.2, 0.3, 0.4 and 0.5) as crystalline structures with cubic Bravais lattice. The Rietveld refinement analysis in **Supplementary Fig. 1** and Goldschmidt's tolerance factor calculation are consistent with each other, confirming all the air-sintered $Pr_{0.5}Ba_{0.5-x}Sr_xFeO_{3-\delta}$ (x = 0, 0.1, 0.2, 0.3, 0.4 and 0.5) as simple perovskite with space group P*m*-3*m*.

$$t \text{ (Goldschmidt Tolerance Factor)} = R_A + R_O / \sqrt{2}\,(R_B + R_O)$$
